# Bottle‐feeding an infant feeding modality: An integrative literature review

**DOI:** 10.1111/mcn.12939

**Published:** 2020-01-06

**Authors:** Judith Kotowski, Cathrine Fowler, Christina Hourigan, Fiona Orr

**Affiliations:** ^1^ Faculty of Health University of Technology Sydney Sydney New South Wales Australia; ^2^ Tresillian Family Care Centres Sydney Sydney New South Wales Australia

**Keywords:** bottle‐feeding, bottle‐feeding equipment, breastfeeding, cues, infant, mechanics, mother

## Abstract

Bottle‐feeding is an infant feeding modality that has been in existence since ancient times, and currently, a significant number of infants are being fed via a bottle with either breastmilk or formula. Although research on bottle‐feeding has continued, it exists in fragmented, often small studies that focus on singular aspects of feeding an infant using a bottle, with limited information on the bottle‐feeding act. Systems theory was the approach used to define the act of bottle‐feeding and identify the parts within this act. Health databases were searched using MeSH terms. A summary of the studies are included. The findings of this review revealed that healthy term bottle‐feeding infants use similar tongue and jaw movements, can create suction and sequentially use teat compression to obtain milk, with minimal differences in oxygen saturation and SSB patterns, when compared with breastfeeding infants. Bottle and teat characteristics were revealed to affect infant feeding and milk intake. An infant's milk intake during feeding was shown to have a strong association with the interaction between the infant and parent/caregiver. With the issue of who controls the feed, mother or infant, likely to affect an infant's ability to self‐regulate their milk intake. Redefining bottle‐feeding as a holistic system identifies the interrelationship of the various parts which will improve the understanding of the reciprocal nature of infant feeding. To optimize bottle‐feeding outcomes, further research is required on parents' and health professionals' knowledge and understanding of the parts within the act of bottle‐feeding.

**Key messages**

- While breastfeeding is a global priority, a significant number of infants are fed using a bottle.
- There is limited quality information for parents/caregivers and health professionals on the act of bottle‐feeding.
- The anatomical actions used by healthy term infants are similar when breast or bottle‐feeding.
- Teat flow rates affect an infant's sucking pattern and milk intake. There is significant variability within the brand and classification of the teat flow rate.
- The anatomical actions used by healthy term infants are similar when breast or bottle‐feeding.
- Infants are interactive partners in the process of bottle‐feeding.

## 1 INTRODUCTION

Infant feeding is essential for survival. The primary infant feeding modalities for infants under 6 months of age are breastfeeding and bottle‐feeding. The ideal feeding modality for mothers and infants is breastfeeding. Global initiatives aim to enhance uptake and duration of breastfeeding (World Health Organization (WHO) & United Nations Children's Fund (UNICEF), 2003). There is no dispute as to the importance of breastfeeding. However, bottle‐feeding plays a significant role in infant nutrition (National Health & Medical Research Council, 2012; World Health Organization (WHO), 1981). Globally, 59% of infants (World Health Organization (WHO), 2018) and around 85% of infants within Australia (Australian Institute of Health and Welfare, 2011) by 5 months of age are being fed with either breastmilk or formula using bottles.

Breastfeeding guidelines and recommendations discuss strategies to assist the dyad if problems arise during the establishment and maintenance of breastfeeding (National Health & Medical Research Council, 2012). Topics covered in the guidelines and policies include positioning and attachment of the infant on the breast, mother's position, milk transfer and production, feeding a baby to their need, normal infant behaviour, and everyday problems (National Health & Medical Research Council, 2012; World Health Organization (WHO), 2017).

Bottle‐feeding guidelines and recommendations, however, tend to focus on aspects of health and safety (National Health & Medical Research Council, 2012; WHO, 1981). Bottle‐feeding advice concentrates on more procedural recommendations: cleaning and sterilizing of feeding equipment, correct preparation of formula, and the storage and transport of formula (United Nations Children's Fund (UNICEF) UK, 2015). Although these guidelines are indeed important, what appears to be lacking is the consideration of bottle‐feeding as a holistic system and how to optimally feed infants when using a bottle.

Previous research indicates there are differences in the practice of breastfeeding and bottle‐feeding and mothers' behaviours (Crow, Fawcett, & Wright, 1980). Parents and health practitioners who support them are faced with a myriad of decisions in the application of bottle‐feeding. This review considers the importance of infant positioning and attachment on the bottle, differences between bottles and teats, and the parent infant feeding interaction.

An integrative review of the empirical literature will investigate these aspects of bottle‐feeding. General systems theory is the theoretical construct of the review, in recognition that bottle‐feeding is a system of interdependent parts. Thus, no one aspect is independent. The majority of research on the topic of bottle‐feeding has investigated the various aspects. This review will consider these within the context of a holistic system and how to support optimal feeding when using a bottle.

## 2 METHODS

This review aims to provide “a new way of thinking about …” (Torraco, 2016, p. 412), the act of bottle‐feeding as an infant‐feeding modality. An integrative literature review methodology is well‐suited for this review as it is a form of research structured around existing literature that intends to answer a specific research question (Torraco, 2016). The scope of this review considers bottle‐feeding as a system (Bertalanffy, 1972). The successful functioning of a system relies upon the contribution of interrelated parts (Broderick, 1993). Fundamental aspects of bottle‐feeding include, how infants obtain milk from a bottle, how bottle‐feeding equipment influences milk delivery to the infant, and how the infant's milk intake may be affected by parent–infant communication during bottle‐feeding.

**Figure 1:**
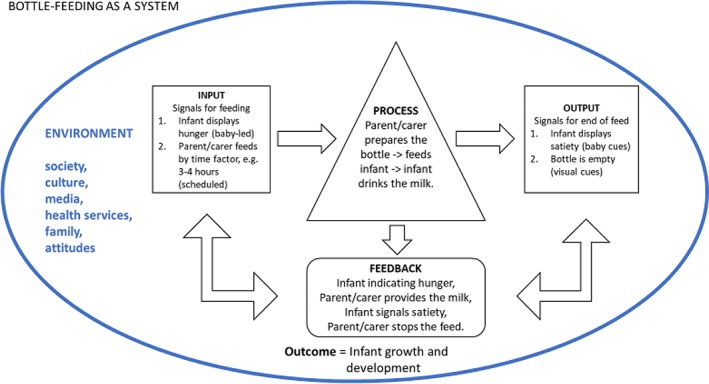
The theoretical construct of bottle‐feeding as a system

There are external influences within the environment that impact on bottle‐feeding, society, culture, media, health services, family, and attitudes, and these variables lay outside the scope of this review. The review's focus is to deepen our understanding of the physiological and functional parts of the bottle‐feeding act. The graphical representation in Figure 1 shows bottle‐feeding as a system (diagram adapted from http://ric357.ru/ludwig-von-bertalanffy-general-system-theory-78/).

### 2.1 Inclusion criteria

Studies with a focus on healthy full‐term infants under 6 months of age, fed via a bottle with either breast milk or formula, were eligible for inclusion. Infants under 6 months were the criterion considered appropriate because the maturation of an infant's oral‐motor function, developmental changes, and primitive feeding reflexes (tongue thrust) disappear around 6 months when an infant starts solid feeding. Studies with a focus on the mechanics of how infants bottle‐feed, the mechanics of bottles and teats as a milk delivery system, and the mother/carer's actions during the bottle‐feeding interaction, were eligible. Sex and ethnicity of the bottle‐feeding dyad was not a limiting factor for inclusion.

### 2.2 Exclusion criteria

Studies that focused on preterm infants, breastfeeding outcomes only, infants over 6 months of age, bottle‐feeding relating to specific medical conditions, and infants with ongoing medical issues, were excluded. These variables have the potential to affect an infant's oral‐motor function, thus outside the scope of the review. Studies that investigated the mother's attitudes, experiences, and choice of feeding modality also did not align with the scope of the review.

### 2.3 Search strategy

**Figure 2:**
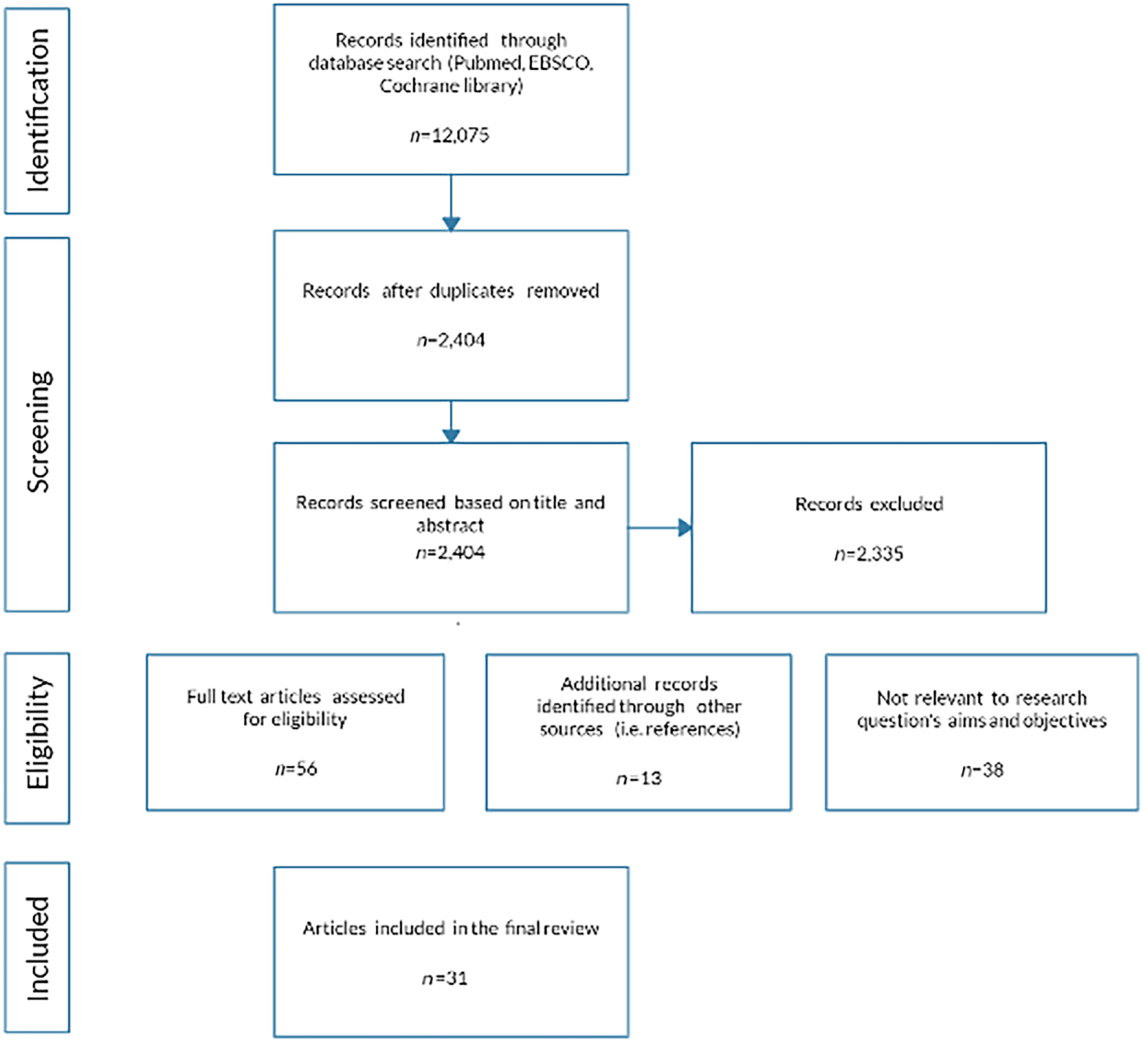
PRISMA diagram bottle‐feeding search results

An initial search of the literature was conducted before October 2019 using the term bottle‐feeding resulting in 12,075 articles. The databases searched include CINAHL, PsycINFO, Medline, ProQuest, and Scopus. MeSH terms, bottle‐feeding, and infant, and anatomy, and physiology, and bottle‐feeding equipment, and communication and cues, were then inserted in the search criteria to align with the scope of this review. Articles were limited to English, peer‐reviewed studies. The articles for review numbered 2,404, once duplicates were removed. The title and abstracts were scrutinized using the inclusion and exclusion criteria, 56 articles were selected. A search of these articles' references was undertaken to reveal a further 13 relevant studies. A full‐text screen was carried out on the 69 retrieved articles; of these, 38 articles did not meet the inclusion criteria. The final 31 articles are in this review. The PRISMA flowchart (Buck, 2019) illustrates the search process (refer to Figure 2).

### 2.4 DATA ANALYSIS

The studies were grouped according to the fundamental aspects of bottle‐feeding. Eight studies focused on the mechanics of how infants obtain milk from bottles. Thirteen studies focused on the characteristics of how bottles and teats affect milk delivery to the infant. Ten studies examined how communication and cues during the feeding interaction affect an infant's milk intake. The table of evidence presents a tabulation of the studies, refer to Table 1.

**Table 1:** Table of evidence

Author/Date/Context	Aim/objective/purpose	Sample Criteria/Size	Method/data gathering	Findings
Mechanics involved in how infants obtain milk from bottles				
Ardran et al. (1958) UK & Sweden	Record and understand how babies obtain milk from a bottle	English, 15 infants; Swedish, 20 infants; full‐term infants Nine lambs + kid goats used a veterinary teat.	Observational, descriptive. Cineradiographic & still films—barium mixed in milk in bottle. Films taken of infants laying on a couch. Infants compared with animal films.	Gravity key in bottle‐feeding; teats too rigid with small hole; expression is main action of feeding; suction can happen with tongue movement; mechanism of swallowing different to adults; disproved theory of feeding and swallowing at the same time. CCAT score: 55% Prelim, 4; Intro, 1; Design, 2; Sampling, 2; Data collection, 4; Ethics 4; Results, 3; and Discussion, 3.
da Costa et al. (2010) Netherlands	Study sucking patterns in healthy term infants and to describe the age‐specific variations.	30 healthy, term infants.	Longitudinal. Recorded five to seven feeding episodes for each infant. Assessed off‐line with the Neonatal Oral‐Motor Assessment Scale (NOMAS). Analysed the first 2 min of feed.	Twenty‐seven infants had normal sucking pattern. Abnormal sucking patterns were observed in 23 of 171 feeding episodes (14%). Ten infants displayed arrhythmical sucking pattern—seen more in bottle‐feeding—up to 10 weeks of age. Variables of infants discussed.CCAT score: 83% Prelim, 5; Intro, 5; Design, 4; Sampling, 4 Data collection, 4; Ethics, 4; Results, 4; and Discussion, 3.
Goldfield et al. (2006) USA	Compare the coordination of sucking, swallowing, and breathing during breastfeeding and bottle‐feeding and examine relationship between oxygen saturation & coordination.	Thirty‐six healthy term infant birth. Mother's breast‐feeding exclusively for 4‐6 weeks before introducing a bottle.	Prospective, Infants own control. Two different bottle systems, soft‐walled (Playtex) vs hard‐walled bottle system (Avent). Pressure catheter on nipple and in teat, microphone on throat, pulse oximeter & respiratory band recorded 3‐4 min of feeding. Circular statistics.	Breastfeeding non‐random swallows, coordinated suck–swallow–breathe (SSB) with high oxygen saturation. Bottle‐feeding system 1 (Playtex)—decrease in swallowing, otherwise like breastfeeding SSB, and oxygenation. Bottle‐feeding system 2 (Avent)—increase & variability in swallowing with reduced oxygenation. Grant Playtex products. CCAT score: 83% Prelim, 4; Intro, 5; Design, 4; Sampling, 4; Data collection, 4; Ethics, 4; Results, 4; Discussion, 4.
Moral et al. (2010) Spain	Assess mechanics of feeding movements in breastfeeding, bottle‐feeding and mixed feeding.	359 Healthy term infants. 62 breastfeeding, 62 bottle‐feeding; 110 mixed feeding infants.	Descriptive, cross‐sectional, randomized open cross‐over field trial. Mixed feeding infants' own control. Feed observed, timed, recorded for >5 min. Sucks/min counted in first 2 min. Medium flow teat, same brand used.	Exclusively bottle‐fed fewer sucks, same number of pauses as breastfeeding but of longer duration. Statically significant differences of sucking and pauses only small. Sucking pressure and feeding volume not measured. Mixed feeders use both breastfeeding and bottle‐feeding sucking movements. Feeding volume not measured. Sponsored by Roche Diagnostics, S.L. CCAT score: 88% Prelim, 5; Intro, 5; Design, 4; Sampling, 5; Data collection, 4; Ethics, 4; Results, 4; Discussion, 4.
Qureshi et al. (2002) USA	Establish normative data for the development of rhythmic suckle feeding for term infants.	16 bottle‐feeding healthy term infants >2500gms	Observational descriptive. Pharyngeal and nipple pressures recorded 1‐4 days of age and again at 1 month.	With an increase in infant's age swallowing, runs of sucking, milk intake increased. Stability of suck swallow rhythm remained unchanged. Ratio of suck swallow changed to more than 1 suck per swallow with maturation. Individuality a factor in results. CCAT score: 78% Prelim, 5; Intro, 4; Design, 4; Sampling, 3; Data collection, 3; Ethics, 4; Results, 4; Discussion, 4.
Sakalidis et al. (2012) Australia	Hypothesis: When using only vacuum to remove milk from a teat, infants would show safe and well‐coordinated patterns like breastfeeding.	16 healthy full‐term infants without feeding difficulties. Breastfeeding and occasionally bottle‐feeding with EBM.	Observational descriptive Ultrasound Recordings of intraoral vacuum, tongue movement, respiration, oxygen saturation, heart rate for entire feed by a computerized data collection system.	Oxygen saturation, heart and respiratory rate with suck swallow breathing patterns being the same for infant's breastfeeding and using the experimental teat. Infants compressed the teat during the latter part of the feed. Limitation discussed. Grant Medula. CCAT score: 83% Prelim, 4; Intro, 4; Design, 4; Sampling, 4; Data collection, 5; Ethics, 4; Results, 4; Discussion, 4.
Taki et al. (2010) Japan	Clarify the differences in longitudinal sucking performance changes in feeding behaviour in infants 1‐6 months of age by comparing breast and bottle‐feeding.	16 breast‐fed, eight bottle‐fed healthy term infants. All infants had fed by both feeding methods.	Observational descriptive Data gathered at 1, 3, 6 months of age. Breastfed infants test weighed. Measurements of vacuum via pressure transducer. Variables relate to sucks, time and efficiency of feeding and sucking pressure. Infants fed with their usual teat (Pigeon) in a semi‐upright supine position.	No significant difference in anthropometric measurements, sucking pressure and efficiency, and milk intake between breast and bottle feeders. The total feeding time, duration per sucking burst, total length of resting time (shortened with age) was postulated as due to milk flow patterns of breast and bottle‐feeding. Maturation appeared to influence feeding efficiency. Discussed differences between breast and bottle‐feeding. Sucking performance varied depending upon which part of feed measured. CCAT score: 75% Prelim, 4; Intro, 4; Design, 4; Sampling, 3; Data collection, 4; Ethics, 4; Results, 4; Discussion, 3
Weber et al. (1986) England	Explain the organization of events that occur inside the baby's mouth during a feed.	Six breastfed Six bottle‐fed	Observational, descriptive by ultrasound with respiratory movements recorded. Preformed between 2‐6 days of birth. 15 breastfed, eight bottle‐fed examined to allow six good films of each group.	Difference in suck swallow ratio for breastfed compared to bottle‐fed infants before 4 days to after 4 days – related to milk availability. Breathing synchronized with sucking – function of maturity or experience. Differences for bottle‐fed infants relate to teat characteristics. Older infants showed more coordination of sucking swallowing and breathing. CCAT score: 60% Prelim, 4; Intro, 3; Design, 3; Sampling, 2; Data collection, 3; Ethics, 3; Results, 3; Discussion, 3.
The characteristics of bottles & teats affecting milk delivery to the infant				
Fadavi et al. (1997) USA	Compare the feeding characteristics of four different commercially available nipple units based on shapes and configurations of nipples.	48 healthy term infants, 2 days of age.	Nonprobability sample—randomly assigned to four groups. Feeding session undertaken by researcher. Data acquisition system recorded intraoral pressure, flow, frequency of sucking, work, power, volume of milk per suck, oxygen saturation.	No statistical significance between teats. Teat hole size, pliability impacts milk flow. Breastfed infants breathed within sucking bursts, bottle‐fed breathed before and after bursts. NUK teat lowers work per suck, total number of sucks per volume was higher, total time to feed was longer equates to lower flow. Funded in part by Gerber. CCAT score: 80% Prelim, 5; Intro, 5; Design, 4; Sampling, 3; Data collection, 4; Ethics, 4; Results, 3; Discussion, 4.
Fewtrell et al. (2012) UK	Whether the design of an anti‐vacuum bottle influences milk intake, growth or behaviour.	63 healthy term infants, exclusively breastfeeding or bottle‐feeding with English speaking Mothers.	Randomized trial. Two groups—Bottle “A” partial anti‐vacuum (Avent), Bottle “B” complete anti‐vacuum (Dr Browns). Breast‐feeding reference group. Outcome measures taken at 2, 3, 4 weeks and then 3 months. Diary of infant behaviour at 2 weeks, opinion of bottle and if any breastfeeds.	No difference between groups for, weight gain, milk intake, ear or gastrointestinal infections, colic. Bottle A infants less fussing. Breastfeeding shorter sleep times, with greater feed times. Mothers reported bottle “A” ease of cleaning and assembly compared with “B” bottle parts. Grant from Phillips AVENT. CCAT score: 93% Prelim, 5; Intro, 5; Design, 4; Sampling, 5; Data collection, 5; Ethics, 4; Results, 5; Discussion, 4.
Geddes et al. (2012) Australia	To determine if breastfed infants could remove breast milk from an experimental teat (ET) designed to release milk only when a vacuum is applied.	18 healthy term infants fed expressed breast milk via a bottle. Infants 49 days old (first session) + 56 days old (2^nd^ session). Exclusion = infants unwell, had feeding difficulties, oral anomalies.	Mo measured 24 hr milk supply–attended lab twice, 15 days apart. Submental ultrasound images + intra‐oral vacuum movements recorded simultaneously during 17 breastfeeding infants and 15 infants using the ET teat. Milk removal—infant test weighed.	Confirmation of milk removal from ET by suction, a similar tongue movement to breastfeeding. Discussed vacuum results and scenarios between feeding modalities. Feeding behaviour changes during feed, as does compression and vacuum for both breast‐ and bottle‐feeding. Flow rates appear to influence tongue movement. Clarified nipple and teat position in relation to junction hard and soft palate‐ equal for both when tongue down, closer for ET when tongue up. Grant Medela. CCAT score: 83% Prelim, 4; Intro, 4; Design, 4; Sampling, 4; Data collection, 5; Ethics, 4; Results, 4; Discussion, 4.
Mathew (1990)	To elucidate the role of hole size and thickness in determining milk flow through nipple units during bottle‐feeding.	20 teats were evaluated.	Tests and measurements on size of nipple hole, thickness at tip of nipple, airflow, milk flow was described.	Results confirm previous study—variability of milk flow in teats. Milk flow and airflow relates to teat hole size. Thickness of the teat tip was not seen as significant in milk flow. Postulate, reducing milk flow will have a positive effect on apnea and bradycardia. Limitations and practical recommendations made. CCAT score: 78% Prelim, 5; Intro, 5; Design, 4; Sampling, 4; Data collection, 4; Ethics, N/A; Results, 5; Discussion, 4.
Mathew (1988) USA	Evaluate the flow characteristics of nipple units currently available for use in the neonatal period.	Standard and Nuk teats used. 30 of each type only used once.	Observational descriptive. Mechanical system designed to measure simulated sucks required to empty 120 mL of formula.	Milk flow of teats varied between brands and within brands tested. Difference between standard and Nuk teats. Milk flow linked to hole size and material rigidity. Negative pressure affects milk flow—higher pressure decreased in milk flow. Cross‐cut teat tested with no milk flow. Discusses implication of different teat flow rates. CCAT score: 78% Prelim, 5; Intro, 5; Design, 4; Sampling, 4; Data collection, 4; Ethics, N/A; Results, 5; Discussion, 4.
Nowak et al. (1994) USA	– To determine whether ultrasonography can be used to visualize artificial nipples while an infant is sucking. – To compare differences of the artificial nipple during sucking. – To compare the suck mechanism when feeding from four types of artificial nipples.	35 bottle‐feeding infants. 6‐12 weeks of age. Setting hospital. Conventional shaped teats—10 infants used a Ross teat, seven Playtex, 11 EvenFlo. Seven infants used Nuk orthodontic shaped.	Nonrandomized clinical study using ultrasound. Observations of teat shape, position of the tongue, cheeks, soft palate. Results compared to 16 breast‐fed infants.	Human nipple is more elastic than the four teats tested. Nuk nipple more compressible, different flow rate to other teats. Conventional teats allowed infants a similar suck pattern to breastfeeding infants with vacuum used not compression for milk removal, being a factor identified as like breastfeeding. Implications for practice discussed. Grant Ross Laboratories. CCAT score: 78% Prelim, 4; Intro, 4; Design, 4; Sampling, 3; Data collection, 4; Ethics, 4; Results, 4; Discussion, 4.
Nowak et al. (1995) USA	Compare measurements of length, compressibility and other characteristics of a new tri‐cut teat with a previous study on the human nipple.	15 healthy term, bottle‐fed infants	Observational study by ultrasound imaging of two angles on tri‐cut teat. Measured teat length, compressibility and assessed mouth seal in first 2 min of feeding.	Tri‐cut teat comparable with breastfeeding. Negative pressure in oral cavity like breast. Mouth seal on teat. Teat compressed to oral pharynx, stretched 122.2% of resting length. Grant Johnson & Johnson. CCAT score: 78%Prelim, 4; Intro, 4; Design, 4; Sampling, 3; Data collection, 4; Ethics, 4; Results, 4; Discussion, 4.
Pados et al. (2015)	Test the milk flow rates and variability in flow rates of currently available bottle nipples used in hospitals.	29 nipple types, 10 nipples of each type were tested.	The amount of formula expressed in 1 min by a breast pump. Mean milk flow rate (mL/min) and coefficient of variation were used to compare nipples within brand and within category.	Flow rates varied between teats and among teats of the same type. The designated flow rate of the teat was not always accurate. Brands description of teat characteristics and flow rates. Limitations discussed.CCAT score: 80% Prelim, 4; Intro, 5; Design, 4; Sampling, 5; Data collection, 5; Ethics, N/A; Results, 5; Discussion, 5.
Pados et al. (2016) USA	Test the milk flow rates and variability in flow rates of bottle nipples used after hospital discharge.	26 nipple types – 15 common brands. 10 of each nipple type were tested.	Purposeful teat sampling from common store locations. Used the same data gathering methods as their previous study. Medula breast pump used.	Study methods same as previous study tested teats used in hospital. Confirmed results and found wide variation in milk flow rates between brands and within same teats brands. Discussed the use of breast pump not indicative of infant's ability to use their oral motor function. CCAT score: 80% Prelim, 4; Intro, 5; Design, 4; Sampling, 5; Data collection, 5; Ethics, N/A; Results, 5; Discussion, 5.
Pados et al. (2019) USA	Test the milk flow rates and variability in flow from bottle teats used in hospital and after hospital discharge.	375 individual nipples tested. 10 types used in hospitals, 15 types in the community = 25 types identified. 15 teats of type. Sample size determined by 80% power at level.05.	Same methodology process as previous studies to test latest teats that have entered market since 2015. Compared drip flow rate with suction method to assess flow rate.	Identified milk flow differences between teat brands classified as extra slow, slow flow and standard used in hospitals. Teats used in the community, were significant differences in milk flow between brands. Concluded drip flow rate not a reliable method to assess flow rate especially for non‐vented bottles and single use teats. Limitation and implications for practice discussed. CCAT score: 85% Prelim, 4; Intro, 5; Design, 5; Sampling, 5; Data collection, 5; Ethics, N/A; Results, 5; Discussion, 5.
Salisbury (1975) England	To estimate the volume obtained per suck during bottle‐feeding. To investigate the effect of changing the shape and size of the teat‐hole on suck volume.	Seven full‐term bottle‐fed infants. Medium‐hole teats with either a hole or slot. Two types of bottles used: conventional and a bottle fitted with a valve.	Observational study. Infants randomized to conventional hole or new design slot teat. Infants were then exposed to the other teat. Conventional bottle and valve bottle used with the different teats. Number of sucks counted, and volume of milk calculated by weighing bottles.	Compared the study's results of volume of milk ingested to breastfeeding infants' results. The bottle containing a value counteracts the vacuum build‐up in bottles when infants feed. Milk volume per suck is greater with the slot teat in a vertical position when using the bottle containing a valve. The bottle with a valve appears to be more advantageous for milk flow than teat. CCAT score: 58% Prelim, 3; Intro, 3; Design, 4; Sampling, 3; Data collection, 4; Ethics, 0; Results, 3; Discussion, 3.
Walden and Prendergast (2000) England	Compared the flow rate of a single hole teat with a cross‐cut teat by simulated sucking.	Two teats—cross‐cut and single hole. Five teats in each group.	Observational descriptive study. Volume flow measured by allowing milk to flow via gravity, and pressure measurement via a chamber calibrated to simulate compression by an infant.	The style of teat effects flow rate. No differences between flow rates when measured different pressures. Cross‐cut teats produce a faster flow rate than single hole teats when constant compression is applied. Postulated that infants may change sucking to accommodate flow rate when using cross‐cut teats. Funded Jackel International Ltd. CCAT score: 58% Prelim, 4; Intro, 3; Design, 3; Sampling, 3; Data collection, 4; Ethics, N/A; Results, 3; Discussion, 3.
Wood et al. (2016) USA	Determine range of bottle sizes used and examine the relationship between bottle size and total daily consumption of formula.	378 Healthy term exclusively bottle‐feeding infants at 2 months of age.	Cross‐sectional analysis of survey data from previous multicentre cluster randomized trial investigating childhood obesity. Questionnaire on formula intake in a day.	Larger bottle size equated to more formula consumed. Possible factors for using larger bottle size was ethnicity, age and higher weight of infants. Other variables: size and flow rate of teats need consideration. Recall bias was discussed. CCAT score: 95% Prelim, 5; Intro, 5; Design, 5; Sampling, 5; Data collection, 5; Ethics, 4; Results, 4; Discussion, 5.
Parent–infant communication and cues during the feeding interaction				
Crow et al. (1980) Scotland	The aims: how the frequency of mothers behaviours are related to infant's birth weight during the first week of life and to describe mothers behaviour in terms of infant behaviour during the feed.	Forty mother‐infant dyads with healthy term infants; 21 breastfeeding—12 males, nine females; 19 bottle‐feeding—nine males, 10 females.	Observational study with the dyads being visited seven times from birth until the infant was 6 months of age. Feeding was videotaped, records made of feeding interaction, records of infant's milk intake over a 3‐day period and birth weight. Mother and infant behavioural categories were developed to operationalize the concept of who controls the start, activities during the feed and the end of a feed.	Suggestion that infants lower birth weight affects mothers feeding style. Association between pressure to feed a smaller infant and the less they consumed, longer the feed. Advice within hospitals may be a reason for mother's pressuring behaviour. Infant controls the feed when breastfeeding—regulates milk intake. Mother controls the feed when bottle‐feeding—possibly inhibiting the infant's self‐regulation of milk intake. Limitations and implications for practice discussed. CCAT score: 85% Prelim, 5; Intro, 5; Design, 4; Sampling, 4; Data collection, 5; Ethics, 3; Results, 4; Discussion, 4.
Golen and Ventura (2015) USA	Explored whether “mindless feeding,” or maternal distraction during bottle‐feeding, is associated with greater infant formula/milk intakes and lower maternal sensitivity to infant cues.	28 infants less than 24 weeks of age.	Video recording of feeds assessed by Nursing Child Assessment Feeding Scale.	Mother's distraction during bottle‐feeding coupled with infant characteristics affects milk intake. Larger milk intake is associated with infant's age; mother's sensitivity to infant cues affected by her distraction; infant's lower self‐regulation and higher emotional reactivity is a factor. No clear definition of over or under feeding. Only one feed examined. Limitations and future research discussed. CCAT score: 88% Prelim, 5; Intro, 5; Design, 4; Sampling, 4; Data collection, 4; Ethics, 4; Results, 4; Discussion, 5.
Rybski et al. (1984) USA	To examine sucking behaviours in normal neonates during all feeds over the course of a 24‐hr period and to document maternal‐infant interactions in bottle‐fed infants.	10 normal full‐term female infants 48 hours of age. Males were excluded because of possible effects of circumcision.	Six feeds in 24 hrs video‐taped and timed. Maternal behaviours recorded—tender touching, eye contact, auditory stimulation. Mother instructed on how to feed. Teat drip rate measured. Feeding occurred in the hospital bassinet with infants positioned supine, head elevated to a 45° angle, mother had limited contact during feeding due to infant's position in bassinet.	Mothers behaviours remained constant across the 24 hr. No differences in infant's milk intake at different feeds in the 24 hr. Postulate feeding may not be a variable to measure maternal behaviour. Positioning of infant not a variable. CCAT score: 73% Prelim, 4; Intro, 4; Design, 3; Sampling, 3; Data collection, 4; Ethics, 3; Results, 4; Discussion, 4.
Ventura and Golen (2015) USA	Compare mother's sensitivity and responsiveness to infant cues using two different bottles. Examine mothers' feeding style and effect of bottle type on feeding interaction and infant's intake.	25 dyads formula feeding. Full‐term, no medical conditions	Experimental pilot NCAST ‐ Sensitivity to Cues and Response to Child's Distress subscales and video recordings. Weight measurements for infants and mothers. Questionnaires on demographics, feeding styles.	Opaque weighted bottle greater levels of responsiveness from mothers. Infants consumed less formula using opaque bottle. Mothers accepted either bottle willingly. Hypothesis of visual and weight cues of different affecting mothers' feeding style were supported. Limitation and further research discussed. CCAT score: 85% Prelim, 5; Intro, 5; Design, 4; Sampling, 4; Data collection, 4; Ethics, 4; Results, 4; Discussion, 4.
Ventura and Hernandez (2019) USA	The aim was to expand and test the hypothesis that mothers would exhibit greater sensitivity to their infants' cues and feed their infants less formula or expressed breast milk when feeding from opaque, weighted bottles compared with conventional, clear bottles.	Size = 76 Mother's 18–40 years of age, Infant's < 32 weeks of age or younger predominantly feeding breast milk and/or formula with the dyad having prior experience with bottle‐feeding	A laboratory‐based within‐subject experimental study across two sites. Two feeding observation video‐recorded at same time of day with the different bottles. The Nursing Child Assessment Feeding Scale used for analysis. Weight measurements for infants and mothers. Questionnaires on demographics, feeding patterns and history.	The use of opaque weighted bottle positively affected mothers' sensitivity, and her feeding behaviour. Clarity of infants' cues linked to milk intake using opaque bottle. Infants' demonstrating low clarity of cues no difference in milk intake between the two bottles used. Milk type, breastmilk or formula, no effect on outcomes. Limitation and further research discussed. CCAT score: 95% Prelim, 5; Intro, 5; Design, 4; Sampling, 5; Data collection, 5; Ethics, 4; Results, 5; Discussion, 5.
Ventura and Mennella (2017) USA	Assessment of feeding, individual differences (characteristics) of infants and mothers relate to bottle feeding outcomes—milk volume consumed.	21 dyads bottle feeding, full term, healthy infants	Objective, experimental approach. Video of feeding sessions. Two sessions recorded: 1. mothers fed as normal (ML). 2. Infants to dictate when hungry, feed at infant's pace, end feed when infant displayed fullness (IL). Questionnaires – depicting infant temperament & maternal feeding styles.	Bottle‐feeding outcomes are connected to both mother and infant factors. Characteristics such as race, ethnicity, and obesity influence feeding practices. Suggests bottle‐feeding does not necessarily promote overfeeding. Bottle‐feeding and responsive feeding does happen. Findings are consistent with other studies. CCAT score: 85% Prelim, 5; Intro, 5; Design, 4; Sampling, 4; Data collection, 4; Ethics, 4; Results, 4; Discussion, 4.
Shloim et al. (2017) Israel & UK	Explore infant communication cues during milk feeding and hypothesize that feeding cues vary by feeding mode.	27 infants. 13 breastfeeding 14 bottle‐feeding. Sample from previous study on eating behaviours in pregnancy.	Comparative study. Feeding sessions filmed, frequency of cues recorded at start, middle and end of feed. NCAST list of engagement (hunger) & disengagement (satiety) cues were used. Demographic data.	More disengagement cues recorded. Hunger cues frequent at beginning with satiety cues at end of feed. Breastfeeding infants signaled more than bottle‐feeding infants. No differences in length of feeding between breast and bottle‐feeding. Recommendation made. Limitations discussed. Findings support the author's hypothesis. Funded ‐ Educational award Danone. CCAT score: 80% Prelim, 4; Intro, 4; Design, 4; Sampling, 4; Data collection, 4; Ethics, 4; Results, 4; Discussion, 4.
Ventura et al. (2019)	Explore variability in, and correlates of, infant clarity of cues during feeding interactions.	86 mother‐infant dyads. Infants full‐term, healthy, approximately 15‐16 weeks of age. 53% females. 48 exclusively breastfed ‐breast and bottle 13 breast and formula 25 exclusively formula	Cross‐sectional study, secondary analysis. NCAST feeding interaction video recorded. Questionnaires ‐ demographics, feeding history, feeding styles, infant temperament, and eating behaviours.	Clarity of cues not associated with infant sex, age, temperament, or eating behaviours. Maternal sensitivity and responsive feeding style associated with infant's clarity of cues. Greater the infant's weight and formula feeding associated with lower clarity of infant's cues. Recommendation made. Limitations discussed. CCAT score: 95% Prelim, 5; Intro, 5; Design, 4; Sampling, 4; Data collection, 5; Ethics, 5; Results, 5; Discussion, 5.
Whitfield and Ventura (2019) Canada	Assess maternal responsiveness to infant cues during milk feeding differing by feeding modality. Also, to quantify infant satiation cues by feeding mode.	Nine mother‐infant dyads, Infants less than 6 months of age. Breast milk used in bottles.	Exploratory cross‐sectional (pilot) study. NCAST in‐home, two sessions video recorded, one breastfeeding and one bottle‐feeding, coding software for satiation cues. Questionnaires – demographics	Mothers were more sensitivity to infant cues when breastfeeding than when bottle‐feeding their EBM. Postulate infants have an active role when breastfeeding and not when bottle‐feeding. No difference in number of infant's satiation cues or activity by feeding mode. Limitations discussed; recommendations made. CCAT score: 93% Prelim, 5; Intro, 5; Design, 4; Sampling, 4; Data collection, 5; Ethics, 4; Results, 5; Discussion, 5.
Wright et al. (1980) Scotland	Explore who has control, mother or infant, when feeding, and differences between breast and bottle feeding behaviour and patterns.	132 bottle‐feeding dyads and, 58 breastfeeding dyads <7 days in post‐natal ward. Home visits at 1 and 2 months	Exploratory ethological study. Food diaries over 3 days, completed on day 3, 4, and 5 days of age. Video recording of feeding sessions and recording of behaviour categories during a feed as per Crow et al. (1980). Rate changes of sucking and diurnal variations in milk intake at 1 week, 1 and 2 months were documented. Description of behaviour categories used.	Patterns and behaviours of breastfed infants differ from bottle‐feed infants. Postulate, breastfeeding infants – control ‐ learn to regulate hunger and milk intake depending upon time between feeds and determine the pace and duration of the feed. Breastfeeding mother's play a more passive role in an infant's milk intake. Whereas, mothers have more control of bottle‐feeding infant's intake. Bottle‐fed infants have regular feed times and milk intake regardless of time between feeds. CCAT score: 90% Prelim, 5; Intro, 4; Design, 4; Sampling, 4; Data collection, 4; Ethics, 4; Results, 5; Discussion, 5.

Quality assessment and to reduce bias in the analysis of the studies, the Crowe Critical Appraisal Tool (CCAT) was used (Crowe, Sheppard, & Campbell, 2012). The CCAT tool, a validated method for appraising different research designs, provides a reliable and consistent method for reducing bias in the review. A study is broken down into eight categories, with each category given a score out of 5, a possible total of 40 (100%) for the entire study. These scores illustrate the strengths, reproducibility, replicability, and the weakness of the individual studies (Crowe, 2013). A consensus was obtained on the CCAT scores for 10 of the 31 studies by two of the authors to provide consistency and accuracy to the quality assessment process. The final CCAT scores are in Table 1.

## 3 RESULTS

### 3.1 Study characteristics

As detailed in the table of evidence, there were nine countries represented. Two studies were a joint venture in the United Kingdom and Sweden, and Israel and the United Kingdom. The majority of research designs for the studies were quantitative, using longitudinal, cross‐sectional, observational, and descriptive data, with one exploratory ethological qualitative study. No systematic reviews met the inclusion criteria for this review. A comparison between bottle‐feeding and breastfeeding results was a frequent occurrence. Most studies used convenience sampling consisting of small sample sizes, with the mother identified as the primary caregiver. Health facilities were the main settings for the studies that assessed infants in the first week of life, and the study design required the use of specific medical equipment. Ethical issues were generally lower on the CCAT because of the year the study was published or when ethics approval was not applicable.

### 3.2 FINDINGS

#### 3.2.1 The mechanics involved in how infants obtain milk from bottles

There is a strong association with an infant's age post‐birth and how they obtain milk safely and effectively (Ardran, Kemp, & Lind, 1958; da Costa et al., 2010; Goldfield, Richardson, Lee, & Margetts, 2006; Moral et al., 2010; Nowak, Smith, & Erenberg, 1994; Qureshi, Vice, Taciak, Bosma, & Gewolb, 2002; Sakalidis et al., 2012; Taki et al., 2010; Weber, Woolridge, & Baum, 1986). The mechanisms involved are complex. They relate to the action of the tongue and jaw, suction and compression, and the coordination of sucking, swallowing, and breathing (da Costa et al., 2010; Fadavi, Punwani, & Vidyasagar, 1997; Moral et al., 2010; Weber et al., 1986).

The infant's tongue was identified as playing a significant role in milk transfer when breast‐ or bottle‐feeding (Ardran et al., 1958; Weber et al., 1986). The studies that viewed an infant's feeding by either x‐ray or ultrasound used different terminologies to characterize this tongue and jaw action (Ardran et al., 1958;Geddes et al., 2012 ; Weber et al., 1986). For example, in the seminal study by Ardran et al. (1958), the term “peristaltic” was used to describe the movement of the pharyngeal wall. Their summary discussed compression and squeezing of the teat by the tongue, along with suction, gravity, and teat hole size being factors influencing milk transfer when bottle‐feeding (Ardran et al., 1958). Ultrasound studies used the same terms, peristaltic and piston‐like, yet contradicted which term is applied to which feeding modality (Geddes et al., 2012; Weber et al., 1986). Breast‐ and bottle‐feeding infants use “ … a squeezing or stripping action … ,” breastfeeding infants “ … appeared to be rolling or peristaltic … ,” and bottle‐feeding infants used a “ … piston‐like or squeezing … ,” action (Weber et al., 1986, p. 22). Geddes et al. (2012, p. 448) refer to the tongue action when breastfeeding as “ … piston‐like … ” creating suction and an “… up and down movement rather than peristaltic …”. The majority of the studies concluded that healthy term bottle‐feeding infants use similar tongue and jaw movements compared with breastfeeding infants when obtaining milk during a feed (Ardran et al., 1958; Geddes et al., 2012; Goldfield et al., 2006; Nowak et al., 1994; Weber et al., 1986).

Several studies debated the primacy of suction and or compression in milk transfer when feeding; with suction being the main element proposed for how breastfeeding infants obtain milk and compression as the main element for bottle‐feeding infants (Geddes et al., 2012; Sakalidis et al., 2012; Weber et al., 1986). Both Geddes et al. (2012) and Sakalidis et al. (2012) used an experimental teat that only released milk by suction, which demonstrated that suction was responsible for milk transfer, not compression. The infants who participated in both studies were breastfeeding and had previously supplemented their feeds with a bottle. It was acknowledged that bottle‐feeding term infants do create suction and sequentially use teat compression (Weber et al., 1986), with gravity being a factor in the transfer of milk (Ardran et al., 1958). Ardran et al.'s (1958) methodology could affect their conclusion that gravity is a significant factor for milk transfer as the infants were lying on their backs, possibly affecting the position of the bottle during the feed. No other studies discussed gravity or mentioned an infant's position or the position of the bottle during the feed as variables for consideration.

Coordination of suck–swallow–breath (SSB) patterns were also viewed as an essential element for successful infant feeding (da Costa et al., 2010; Moral et al., 2010; Qureshi et al., 2002; Sakalidis et al., 2012; Taki et al., 2010; Weber et al., 1986). Breast‐ and bottle‐feeding infants' SSB patterns changed because of an infant's age, with the newborn's reflexive SSB pattern of one or more sucks per swallow to longer sucking bursts as the infant matured (Qureshi et al., 2002; Weber et al., 1986). Therefore, the change in SSB pattern differs depending upon which part of the feed is observed (Taki et al., 2010). Overall, there seem to be minimal differences in oxygen saturation and SSB patterns between healthy term breast‐ and bottle‐feeding infants (Fadavi et al., 1997; Goldfield et al., 2006; Sakalidis et al., 2012; Weber et al., 1986). The studies determined that feeding modality does not necessarily influence SSB patterns.

A change in an infant's SSB when breast‐ or bottle‐feeding was associated with milk availability and milk flow rate (Qureshi et al., 2002; Sakalidis et al., 2012; Weber et al., 1986). It was suggested that bottle‐feeding infant's sucking patterns are dependent upon teat and bottle characteristics (da Costa et al., 2010). Taking into consideration how SSB patterns change during a feed, and how bottles/teats influence milk flow, the findings in the study by da Costa et al. (2010) requires reflection. This study only assessed the first 2 min of an infant feeding and found that bottle‐feeding infants had more arrhythmical sucking (AS) patterns compared with breastfeeding infants. When examining the characteristics of the infants with AS patterns, they were consuming high quantities of milk, had low birth weight, needed medical attention in the first couple of days of life, and were choking and leaking milk when feeding.

#### 3.2.2 The characteristics of bottles and teats affecting an infant's milk intake

Bottle and teat characteristics were revealed to affect infant feeding and milk intake. (Ardran et al., 1958; da Costa et al., 2010; Goldfield et al., 2006; Mathew, 1988; Pados, Park, & Dodrill, 2019; Pados, Park, Thoyre, Estrem, & Nix, 2016; Weber et al., 1986). An infant's milk intake, SSB patterns, and oxygenation were compared with breastfeeding using different anti‐vacuum (vented) bottle systems. The bottle with a collapsible bladder and the vented bottle system displayed similar results to the breastfeeding group (Fewtrell, Kennedy, Nicholl, Khakoo, & Lucas, 2012; Goldfield et al., 2006; Salisbury, 1975).

Milk intake and bottle sizes were investigated with larger sized bottles being associated with an infant consuming an extra 15 kcal/kg of milk (Wood et al., 2016). It was discussed other contributing factors as possibly affecting the use of larger bottles were an infant's growth, responsiveness to infant's feeding cues, and parental feeding style (Wood et al., 2016). However, Ventura and Golen's (2015) pilot study examining contextual cues when bottle‐feeding found no relationship to bottle size and milk intake. Their results implicate both the visual and weight of milk in the bottle, regardless of parenting feeding style, affecting an infant's milk intake.

Studies have reported an infant's milk intake is influenced by teat characteristics (Ardran et al., 1958; Geddes et al., 2012; Mathew, 1990; Nowak et al., 1994; Nowak, Smith, & Erenberg, 1995; Pados et al., 2019; Salisbury, 1975), with milk flow rate to an infant affected by the teat material, shape, hole size, its rigidity and compressibility, the bottle material, and the rigidity and pressure used by the infant (Ardran et al., 1958; da Costa et al., 2010; Goldfield et al., 2006; Mathew, 1990; Nowak et al., 1994; Salisbury, 1975; Walden & Prendergast, 2000). Studies that examined commercially available teats found a wide variation in flow rate between brands, within the same brands and the same labelled teat flow rate (Mathew, 1988; Pados et al., 2016; Pados et al., 2019; Pados, Park, Thoyre, Estrem, & Nix, 2015). The conclusion being the company's labelling and terminology on teat flow rates were confusing and it was challenging to compare flow rates between brands (Pados et al., 2016). Two studies using different methods examined flow rates of cross‐cut teats (Mathew, 1988; Walden & Prendergast, 2000). In the study by Mathew (1988) suction was applied to the cross‐cut teat resulting in no flow. However, Walden and Prendergast (2000) found the cross‐cut teat had a faster flow rate during a compression test compared with single hole teats. This study did describe teat characteristics of material and hole size.

Pados et al. (2019) hypothesized that the variability of flow rates within the same teat range is possibly a reason for infant feeding difficulties. It appears that if milk flow is continuous or too fast, this can affect sucking patterns, with some infants unable to adjust their SSB patterns, causing feeding difficulties (Pados et al., 2015). For instance, the faster a teat flows equates to a larger volume delivered, the infant sucks less, with longer pauses between sucks to allow for swallowing and breathing. The consequences of a fast teat flow rate can manifest as breathing anomalies, drooling, and the possibility of aspiration of milk (Pados et al., 2016). Interestingly, commercial enterprise funded several studies; refer to Table 1.

A common weakness within the studies was a lack of description of teat characteristics. Only three studies provided information on the teat material used: latex/rubber and silicone (Moral et al., 2010; Walden & Prendergast, 2000; Weber et al., 1986). The teat flow rate was described in studies as like breastfeeding, medium flow, or the same flow (Fadavi et al., 1997; Moral et al., 2010; Sakalidis et al., 2012). Teats were named either by brand or shape, with little or no detail given on their characteristics (Fadavi et al., 1997; Mathew, 1988; Moral et al., 2010; Nowak et al., 1995; Taki et al., 2010). It is unfortunate that because of teat characteristics, particularly the teat flow rate, discussed in most studies as affecting an infant's sucking and milk intake. The information on the teat flow rate as being inconsistent and too fast may influence some studys' conclusions.

#### 3.2.3 Parent–infant communication and cues during the feeding interaction

An infant's milk intake during feeding has a strong association to the interaction between the infant and parent/caregiver (Crow et al., 1980; Golen & Ventura, 2015; Shloim, Vereijken, Blundell, & Hetherington, 2017; Ventura & Golen, 2015; Ventura & Mennella, 2017; Ventura, Sheeper, & Levy, 2019; Wright, Fawcett, & Crow, 1980). Rybski, Almli, Gisel, Powers, and Maurer (1984) found that a mother's interactions did not change over 24 hr, nor did they affect an infant's milk intake. However, the methodology of this study could be a factor for this finding. Bottle‐feeds were scheduled and observed in a hospital setting, infants were bottle‐fed in a bassinet, mothers were instructed on how to feed their infant and only held their infant to burp them.

Other studies explored bottle‐feeding mother's interactions and linked her feeding style to an infant's milk intake (Ventura et al., 2019; Ventura & Golen, 2015; Ventura & Hernandez, 2019; Ventura & Mennella, 2017). There was an agreement in these studies that when a mother has a pressuring feeding style, the infant is encouraged to consume more milk, and with a restrictive feeding style, the infant has lower milk intake. Mothers with either a pressuring or restrictive feeding style were found to be more responsive to their infant's cues when they could not see or feel the amount of milk in a bottle (Ventura & Golen, 2015; Ventura & Hernandez, 2019). If the mother is distracted during the feeding interaction, she is less sensitive and can miss the infant's cues, which can contribute to under or overfeeding of the infant (Golen & Ventura, 2015).

Studies that explored maternal responsiveness and infant feeding cues found that bottle‐feeding infants actively engage in and reciprocate responses of the mother allowing the infant to be an active participant in the feeding interaction (Ventura et al., 2019; Ventura & Mennella, 2017). However, breastfeeding infants were identified by Shloim et al. (2017) as displaying more cues of hunger and satiety than bottle‐feeding infants. Suggesting this is because of the infant being an active participant when breastfeeding and passive when bottle‐feeding. Whitfield and Ventura (2019) explored infant cues with the number of cues found to be similar for breast‐ and bottle‐feeding. The mother's responsiveness to her infant cues was different when comparing her breast‐ and bottle‐feeding interactions.

The methodology was a possible reason for the discrepancy between these studies. Ventura and Mennella (2017) described 11 self‐coded infant behaviours to measure hunger and satiety. Their study explained the interaction and consequences between both mother and infant; with this being the only study to explore infant temperament as a variable impacting upon the feeding interaction. Whitfield and Ventura (2019, p. 483) used the “Caregiver/Parent Child Interaction Feeding Scale,” taken from the Nursing Child Assessment Satellite Training, with data on both infant and mother to illustrate their findings. Ventura et al. (2019) also used the Caregiver/Parent Child Interaction Feeding Scale, relying on two of the subscales—infant's clarity of cues and maternal sensitivity to cues for their study findings. Shloim et al. (2017) was the only study to explore infant communication without assessing the mother's response, possibly affecting her infant's cues during the feeding interaction. The authors cited the “Nursing Child Assessment Teaching Scale” as their basis for defining engagement and disengagement cues for hunger and satiety. They identified 22 out of the 83 feeding cues to use for data collection during the feeding interaction (Shloim et al., 2017, p. 76).

The feeding interaction was also considered to be influenced by infant characteristics, possibly affecting milk intake (Crow et al., 1980; Golen & Ventura, 2015; Ventura et al., 2019; Ventura & Mennella, 2017; Wright et al., 1980). Infants with low regulation and surgency may not be clear in communicating their needs (Golen & Ventura, 2015; Ventura et al., 2019). Crow et al. (1980) suggest an association between bottle‐feeding infants who had a low birth weight and the mothers' control over the feed. This issue of who controls the feed, mother or infant, is likely to affect an infant's ability to self‐regulate their milk intake (Crow et al., 1980; Wright et al., 1980).

A breastfeeding infant has control over starting and stopping feeding and learns to self‐regulate their intake. Self‐regulation of daily feeding patterns by the breastfeeding infant was not the case with bottle‐feeding infants (Wright et al., 1980). Bottle‐feeding infants usually are given the same volume of milk regardless of the time of day or time between feeds (Wright et al., 1980). The bottle‐feeding mother was viewed as having the control over the amount in the bottle, starting and stopping feeding, probably impacting on the infant's learning to self‐regulate (Crow et al., 1980; Whitfield & Ventura, 2019).

## 4 DISCUSSION

This review has examined 31 studies that have provided insight and information on the fundamental aspects of the mechanics of how infants obtain milk from bottles, the characteristics of bottles and teats affecting an infant's milk intake, and parent infant communication during the feeding interaction. When these parts are viewed in isolation, the utility of the information is not being used to its full potential. Looking at this information from a systems perspective, the understanding of the contribution and connection of the parts is a necessity (Bertalanffy, 1972).

Bottle‐feeding as a system requires the infant, the parent/carer, and the bottle‐feeding equipment to contribute to the process. The connections within and between these parts impact the feeding outcome. The infant's contribution to feeding relies upon their maturity and development of their oral feeding skills (Lau, 2016). The infant's oral feeding skills can be supported by the positioning of both infant and bottle during a feed (Kassing, 2002; Ross & Fuhrman, 2015). Kassing (2002) suggests for the infant to control milk flow, they need to be in an upright position with the bottle held horizontally. With the infant's characteristics of age, weight, and temperament influencing their success at communicating their needs during a feed (Golen & Ventura, 2015; Kielbratowska, Kazmierczak, Michalek, & Preis, 2015).

Parent/carer response to their infant's needs during feeding requires an understanding of their infant's communication. However, a recent systematic review of infant feeding interventions discussed limited evidence on parent/caregiver knowledge concerning responsive feeding practices (Matvienko‐Sikar et al., 2019). Responsive feeding has been suggested as a learned behaviour between the dyad and is a reciprocal activity (Appleton et al., 2018; Oxford, & Findlay, D. (Eds.)., 2015; Ventura, 2018). Global infant feeding guidelines now advocate responsive feeding as a strategy to address milk intake and possible long‐term effects of over‐feeding (United Nations Children's Fund (UNICEF) UK, 2016), with milk delivery via bottles and teats linked to milk intake.

Bottle‐feeding equipment has evolved in design because of the availability of new materials and research on how infants bottle‐feed (Mathew, 1991; Pados et al., 2019; Ventura, 2018). However, commercial enterprise governs the information on bottle and teat characteristics, and there is no required standard to adhere to in the marketing nor labelling of these products (Dowling & Tycon, 2010; Pados et al., 2016). There are significant inconsistencies between the labelling of teats and actual performance (Pados et al., 2019), leaving parents and the health professionals that support them in a quandary when making decisions on appropriate bottle‐feeding equipment.

The integration of the above information will allow strategies to optimize outcomes for both infants and families. For example, information to parent/carers on responsive feeding, how to maximize an infant's oral feeding skills, and the bottle‐feeding technique suggested by Kassing (2002), could be a strategy to offset the variability of teat flow rates; the role of gravity, enabling an infant's to control their milk intake (self‐regulation); and parental responsiveness during the feeding interaction.

**Figure 3:**
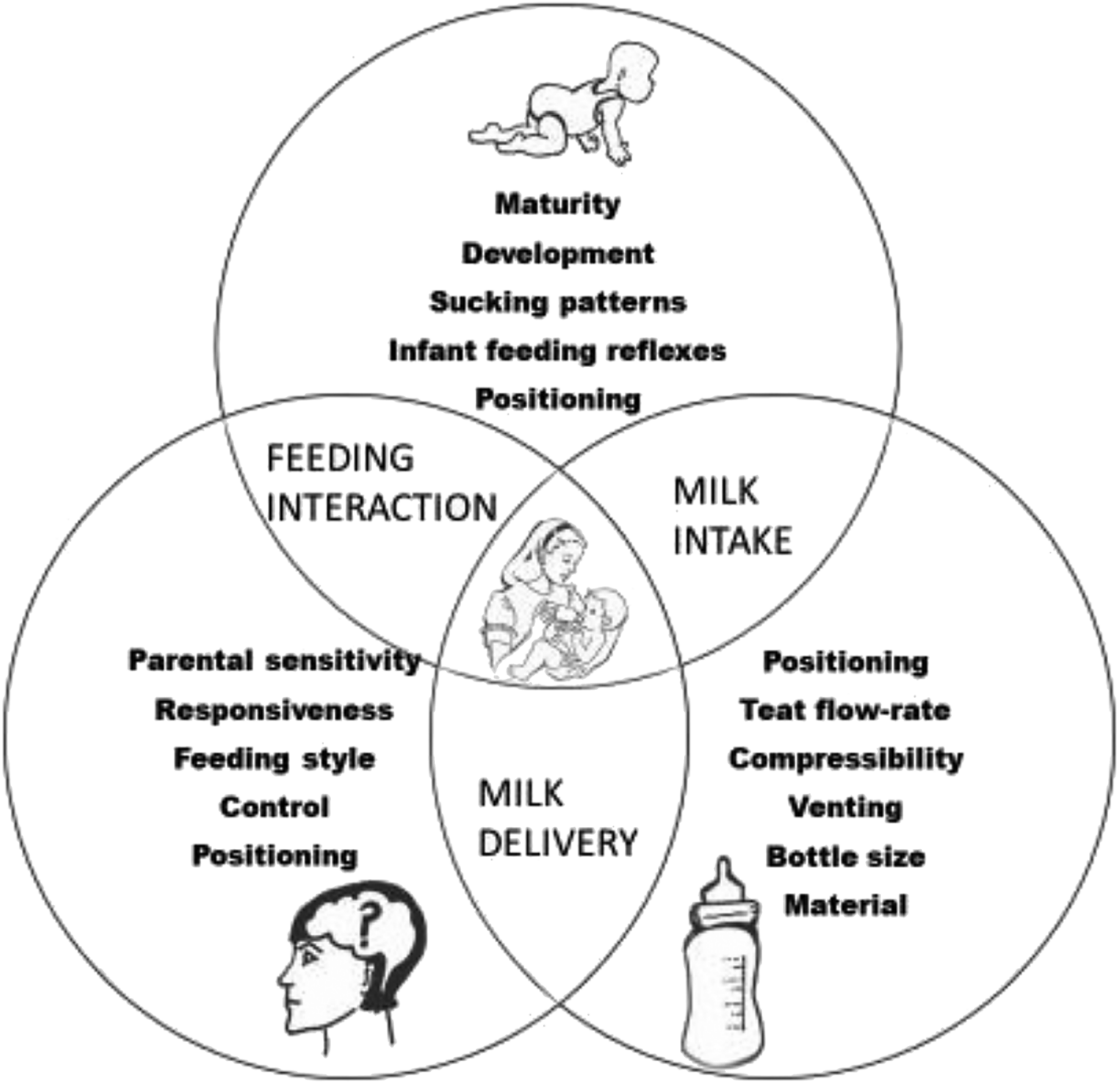
Model of the reconceptualization of the act of bottle‐feeding as a system

Optimal bottle‐feeding relies upon parents and health professionals' understanding of how infants obtain milk, how to use their oral feeding skills, how they attach on the bottle, how to position the infant and bottle, characteristics of the bottle‐feeding equipment, and responsive feeding practices. By redefining the act of bottle‐feeding as a holistic system, the interrelationship of these parts will be recognized along with the reciprocal nature of bottle‐feeding; refer to Figure 3.

### 4.1 FUTURE RESEARCH

An understanding of the connection and relationship of these parts will foster the development of possible strategies to assist health professionals when supporting families who choose to use bottles to feed their infant. This review has identified a need for feeding behaviours of men and other carers to be explored, as well as the investigation of the parents'/carers' understanding of responsive feeding practices, ultimately providing a more holistic picture of this phenomena to guide further research and practice.

### 4.2 LIMITATIONS

Only published peer‐reviewed studies were considered, with other forms of related literature not included, possibly affecting the findings. The heterogeneity of the articles reviewed presents challenges when synthesizing and generalizing findings.

## 5 CONCLUSIONS

Systems theory is a unique approach to consider the act of bottle‐feeding, and it is evident that this act is a complex process that is dependent on many variables working together. When one part is out of sync, it will impact on the other parts of the system influencing the outcome. Before strategies to assist the bottle‐feeding dyad can be developed, investigation of parents' and health professionals' knowledge and understanding on the parts within the act of bottle‐feeding needs to occur.

## CONFLICTS OF INTEREST

The authors declared no potential conflicts of interest with respect to this manuscript, authorship, and/or publication of this article.

## CONTRIBUTIONS

JK led the manuscript's concept, draft, analysis, interpretation of the data, and writing of the manuscript, with input from CF and CH. CF, CH, and FO revised the article critically for important intellectual content. All authors have read and approved the final version of this manuscript.

## References

[mcn12939-bib-0001] Appleton, J. , Laws, R. , Russell, C. G. , Fowler, C. , Campbell, K. J. , & Denney‐Wilson, E. (2018). Infant formula feeding practices and the role of advice and support: An exploratory qualitative study. BMC Pediatrics, 18(1), 12 10.1186/s12887-017-0977-7 29368596 PMC5784678

[mcn12939-bib-0002] Ardran, G. M. , Kemp, F. H. , & Lind, J. (1958). A cineradiographic study of bottle feeding. The British Journal of Radiology, 31(361), 11–22. 10.1259/0007-1285-31-361-11 13489213

[mcn12939-bib-0003] Australian Institute of Health and Welfare . (2011, 13th September 2017). 2010 Australian national infant feeding survey: Indicator results. Retrieved from https://www.aihw.gov.au/reports/mothers-babies/2010-australian-national-infant-feeding-survey/data

[mcn12939-bib-0004] Bertalanffy, L. V. (1972). The history and status of general systems theory. The Academy of Management Journal, 15(4), 407–426. Retrieved from. http://www.jstor.org/stable/255139

[mcn12939-bib-0005] Broderick, C. B. (1993). Understanding family process: Basics of family systems theory: Sage.

[mcn12939-bib-0006] Buck . (2019). Prisma (flowchart). Retrieved from https://creately.com/diagram/example/iomm3thh1/Prisma

[mcn12939-bib-0007] da Costa, S. P. , van der Schans, C. P. , Boelema, S. R. , van der Meij, E. , Boerman, M. A. , & Bos, A. F. (2010). Sucking patterns in fullterm infants between birth and 10 weeks of age. Infant Behavior & Development, 33(1), 61–67. 10.1016/j.infbeh.2009.11.007 20060170

[mcn12939-bib-0008] Crow, R. A. , Fawcett, J. N. , & Wright, P. (1980). Maternal behavior during breast‐ and bottle‐feeding. Journal of Behavioral Medicine, 3(3), 259–277. 10.1007/bf00845051 7441727

[mcn12939-bib-0009] Crowe, M . (2013). Crowe Critical Appraisal Tool (CCAT) version 1.4. Retrieved from https://conchra.com.au/wp-content/uploads/2015/12/CCAT-form-v1.4.pdf

[mcn12939-bib-0010] Crowe, M. , Sheppard, L. , & Campbell, A. (2012). Reliability analysis for a proposed critical appraisal tool demonstrated value for diverse research designs. Journal of Clinical Epidemiology, 65(4), 375–383. 10.1016/j.jclinepi.2011.08.006 22078576

[mcn12939-bib-0011] Dowling, D. A. , & Tycon, L. (2010). Bottle/nipple systems. Nursing for Women's Health, 14(1), 61–66. 10.1111/j.1751-486X.2010.01508.x 20137044

[mcn12939-bib-0012] Fadavi, S. , Punwani, I. C. , & Vidyasagar, D. (1997). Mechanics and energetics of nutritive sucking: A functional comparison of commercially available nipples. The Journal of Pediatrics, 130 10.1016/s0022-3476(97)80016-9 9152283

[mcn12939-bib-0013] Fewtrell, M. , Kennedy, K. , Nicholl, R. , Khakoo, A. , & Lucas, A. (2012). Infant feeding bottle design, growth and behaviour: Results from a randomised trial. BMC Research Notes, 5(150), 1–11. 22424116 10.1186/1756-0500-5-150 PMC3328286

[mcn12939-bib-0014] Geddes, D. T. , Sakalidis, V. S. , Hepworth, A. R. , McClellan, H. L. , Kent, J. C. , Lai, C. T. , & Hartmann, P. E. (2012). Tongue movement and intra‐oral vacuum of term infants during breastfeeding and feeding from an experimental teat that released milk under vacuum only. Early Human Development, 88(6), 443–449. 10.1016/j.earlhumdev.2011.10.012 22119233

[mcn12939-bib-0015] Goldfield, E. C. , Richardson, M. J. , Lee, K. G. , & Margetts, S. (2006). Coordination of sucking, swallowing, and breathing and oxygen saturation during early infant breast‐feeding and bottle‐feeding. Pediatric Research, 60, 450–455. 10.1203/01.pdr.0000238378.24238.9d 16940236

[mcn12939-bib-0016] Golen, R. B. , & Ventura, A. K. (2015). Mindless feeding: Is maternal distraction during bottle‐feeding associated with overfeeding? Appetite, 91, 385–392. 10.1016/j.appet.2015.04.078 25953601 PMC4464819

[mcn12939-bib-0017] Kassing, D. (2002). Bottle‐feeding as a tool to reinforce breastfeeding. Journal of Human Lactation, 18(1), 56–60. 10.1177/089033440201800110 11845739

[mcn12939-bib-0018] Kielbratowska, B. , Kazmierczak, M. , Michalek, J. , & Preis, K. (2015). Temperament and the mother‐infant dyad: associations with breastfeeding and formula feeding with a bottle. Infant Mental Health Journal, 36(3), 243–250. 10.1002/imhj.21508 25973840

[mcn12939-bib-0019] Lau, C. (2016). Development of infant oral feeding skills: What do we know? American Journal of Clinical Nutrition, 103(2), 616S–621S. 10.3945/ajcn.115.109603 26791183 PMC4733254

[mcn12939-bib-0020] Mathew, O. P. (1988). Nipple units for newborn infants: A functional comparison. Pediatrics, 81(5), 688–691. Retrieved from. https://www.ncbi.nlm.nih.gov/pubmed/3357729 3357729

[mcn12939-bib-0021] Mathew, O. P. (1990). Determinants of milk flow through nipple units: Role of hole size and nipple thickness. JAMA Pediatrics, 144(2), 222–224. 10.1001/archpedi.1990.02150260102039 2301329

[mcn12939-bib-0022] Mathew, O. P. (1991). Science of bottle feeding. Pediatrics, 119(4), 511–519. 10.1016/s0022-3476(05)82397-2 1919879

[mcn12939-bib-0023] Matvienko‐Sikar, K. , Griffin, C. , McGrath, N. , Toomey, E. , Byrne, M. , Kelly, C. , … Kearney, P. M. (2019). Developing a core outcome set for childhood obesity prevention: A systematic review. Maternal & Child Nutrition, 15(1), e12680 10.1111/mcn.12680 30136417 PMC7199036

[mcn12939-bib-0024] Moral, A. , Bolibar, I. , Seguranyes, G. , Ustrell, J. M. , Sebastiá, G. , Martínez‐Barba, C. , & Ríos, J. (2010). Mechanics of sucking: Comparison between bottle feeding and breastfeeding. BMC Pediatrics, 10(1), 6 10.1186/1471-2431-10-6 20149217 PMC2837866

[mcn12939-bib-0025] National Health & Medical Research Council . (2012). *Infant feeding guidelines: Information for health workers* [N56](pp. 133). Retrieved from https://www.nhmrc.gov.au/guidelines-publications/n56

[mcn12939-bib-0026] Nowak, A. J. , Smith, W. L. , & Erenberg, A. (1994). Imaging evaluation of artificial nipples during bottle feeding. Archives of Pediatrics & Adolescent Medicine, 48 10.1001/archpedi.1994.02170010042008 8143007

[mcn12939-bib-0027] Nowak, A. J. , Smith, W. L. , & Erenberg, A. (1995). Imaging evaluation of breast‐feeding and bottle‐feeding systems. Journal of Pediatrics, 126, S130–S134. 10.1016/s0022-3476(95)90253-8 7776073

[mcn12939-bib-0028] Oxford, M. , & Findlay, D. (Eds.). (2015). NCAST caregiver/parent‐child interaction feeding manual (2nd ed.). Seattle: NCAST Programs, University of Washington, School of Nursing.

[mcn12939-bib-0029] Pados, B. F. , Park, J. , & Dodrill, P. (2019). Know the flow: Milk flow rates from bottle nipples used in the hospital and after discharge. Advances in Neonatal Care, 19(1), 32–41. 10.1097/ANC.0000000000000538 30028734

[mcn12939-bib-0030] Pados, B. F. , Park, J. , Thoyre, S. , Estrem, H. , & Nix, W. (2016). Milk flow rates from bottle nipples used after hospital discharge. The American Journal of Maternal/Child Nursing, 41(4), E15–E16. 10.1097/NMC.0000000000000271 PMC5033656 27008466

[mcn12939-bib-0031] Pados, B. F. , Park, J. , Thoyre, S. M. , Estrem, H. , & Nix, W. B. (2015). Milk flow rates from bottle nipples used for feeding infants who are hospitalized. American Journal of Speech‐Language Pathology, 24(4), 671–679. 10.1044/2015_AJSLP-15-0011 26172340 PMC4698468

[mcn12939-bib-0032] Qureshi, M. A. , Vice, F. L. , Taciak, V. L. , Bosma, J. F. , & Gewolb, I. H. (2002). Changes in rhythmic suckle feeding patterns in term infants in the first month of life. Developmental Medicine and Child Neurology, 44(1), 34–39. 10.1111/j.1469-8749.2002.tb00256.x 11811648

[mcn12939-bib-0033] Ross, E. , & Fuhrman, L. (2015). Supporting oral feeding skills through bottle selection. Perspectives on Swallowing and Swallowing Disorders (Dysphagia), 24, 50–57.

[mcn12939-bib-0034] Rybski, D. A. , Almli, C. R. , Gisel, E. G. , Powers, J. , & Maurer, M. (1984). Sucking behaviors of normal 3‐day‐old female neonates during a 24‐hr period. Developmental Psychobiology, 17(1), 79–86. 10.1002/dev.420170107 6698312

[mcn12939-bib-0035] Sakalidis, V. S. , McClellan, H. L. , Hepworth, A. R. , Kent, J. C. , Lai, C. T. , Hartmann, P. E. , & Geddes, D. T. (2012). Oxygen saturation and suck‐swallow‐breathe coordination of term infants during breastfeeding and feeding from a teat releasing milk only with vacuum. International Journal Of Pediatrics, 1–10. 10.1155/2012/130769 PMC3398629 22844300

[mcn12939-bib-0036] Salisbury, D. M. (1975). Bottle‐feeding: Influence of teat hole size on suck volume. The Lancet, 655–656. 10.1016/s0140-6736(75)91759-6 47081

[mcn12939-bib-0037] Shloim, N. , Vereijken, C. , Blundell, P. , & Hetherington, M. M. (2017). Looking for cues—Infant communication of hunger and satiation during milk feeding. Appetite, 108, 74–82. 10.1016/j.appet.2016.09.020 27647500

[mcn12939-bib-0038] Taki, M. , Mizuno, K. , Murase, M. , Nishida, Y. , Itabashi, K. , & Mukai, Y. (2010). Maturational changes in the feeding behaviour of infants—A comparison between breast‐feeding and bottle‐feeding. Acta Paediatrica, 99(1), 61–67. 10.1111/j.1651-2227.2009.01498.x 19839957

[mcn12939-bib-0039] Torraco, R. J. (2016). Writing integrative literature reviews: Using the past and present to explore the Future. 15 10.1177/1534484316671606

[mcn12939-bib-0040] United Nations Children's Fund (UNICEF) UK . (2015). Infant formula and responsive bottle feeding. Retrieved from https://www.unicef.org.uk/babyfriendly/baby-friendly-resources/leaflets-and-posters/simple-formula-guide-for-parents/

[mcn12939-bib-0041] United Nations Children's Fund (UNICEF) UK . (2016). Responsive feeding: Supporting close and loving relationships. Retrieved from https://www.unicef.org.uk/babyfriendly/wp-content/uploads/sites/2/2017/12/Responsive-Feeding-Infosheet-Unicef-UK-Baby-Friendly-Initiative.pdf

[mcn12939-bib-0042] Ventura, A. K. (2018). Bottle‐feeding: Perceptions, practices, and health outcomes. New York: Nova Science Publishers, Inc.

[mcn12939-bib-0043] Ventura, A. K. , & Golen, R. P. (2015). A pilot study comparing opaque, weighted bottles with conventional, clear bottles for infant feeding. Appetite, 85, 178–184. 10.1016/j.appet.2014.11.028 25445988 PMC4309547

[mcn12939-bib-0044] Ventura, A. K. , & Hernandez, A. (2019). Effects of opaque, weighted bottles on maternal sensitivity and infant intake. Maternal & Child Nutrition, 15(2), 1–9. 10.1111/mcn.12737 PMC7199074 30345622

[mcn12939-bib-0045] Ventura, A. K. , & Mennella, J. A. (2017). An experimental approach to study individual differences in infants' intake and satiation behaviors during bottle‐feeding. Childhood Obesity, 13(1), 44–52. 10.1089/chi.2016.0122 27788024 PMC5278825

[mcn12939-bib-0046] Ventura, A. K. , Sheeper, S. , & Levy, J. (2019). Exploring correlates of infant clarity of cues during early feeding interactions. Journal of the Academy of Nutrition and Dietetics, 119(9), 1452–1461. 10.1016/j.jand.2019.03.014 31153959 PMC6710109

[mcn12939-bib-0047] Walden, E. , & Prendergast, J. (2000). Infant feeding: Comparison of flow rates of holes versus cross‐cut teats for bottle‐fed babies. Professional Care of Mother and Child, 10(1), 7–8. Retrieved from. https://www.lib.uts.edu.au/goto?url=http://search.ebscohost.com/login.aspx?direct=true&db=cin20&AN=107102168&site=ehost-live 11013566

[mcn12939-bib-0048] Weber, F. , Woolridge, M. W. , & Baum, J. D. (1986). An ultrasonographic study of the organisation of sucking and swallowing by newborn infants. Developmental Medicine and Child Neurology, 28, 19–24. 10.1111/j.1469-8749.1986.tb03825.x 3512348

[mcn12939-bib-0049] Whitfield, K. C. , & Ventura, A. K. (2019). Exploration of responsive feeding during breastfeeding versus bottle feeding of human milk: A within‐subject pilot study. Breastfeeding Medicine, 14(7), 482–486. 10.1089/bfm.2019.0069 31188021

[mcn12939-bib-0050] Wood, C. T. , Skinner, A. C. , Yin, H. S. , Rothman, R. L. , Sanders, L. M. , Delamater, A. M. , … Perrin, E. M. (2016). Association between bottle size and formula intake in 2‐month‐old infants. Academic Pediatrics, 16(3), 254–259. 10.1016/j.acap.2015.08.001 26525989 PMC4808476

[mcn12939-bib-0051] World Health Organization (WHO) (1981). International code of marketing of breastmilk substitutes. Geneva, Switzerland: World Health Organisation Retrieved from. http://www.who.int/nutrition/publications/infantfeeding/9241541601/en/

[mcn12939-bib-0052] World Health Organization (WHO) (2018). Implementation guidance: protecting, promoting and supporting breastfeeding in facilities providing maternity and newborn services – the revised Baby‐friendly Hospital Initiative. Geneva: World Health Organisation.

[mcn12939-bib-0053] World Health Organization (WHO), & United Nations Children's Fund (UNICEF) . (2003). Global strategy for infant and Young child feeding. Retrieved from http://www.who.int/maternal_child_adolescent/documents/9241562218/en/

[mcn12939-bib-0054] World Health Organization (WHO) (2017). Protecting, promoting and supporting breastfeeding in facilities providing maternity and newborn services. Nutrition Retrieved from. https://www.who.int/nutrition/publications/guidelines/breastfeeding-facilities-maternity-newborn/en/ 29565522

[mcn12939-bib-0055] Wright, P. , Fawcett, J. , & Crow, R. (1980). The development of differences in the feeding behaviour of bottle and breast fed human infants from birth to two months Behavioural Processes, 5, 1‐20. 10.1016/0376-6357(80)90045-5 24925154

